# Inpatient Telemedicine and New Models of Care during COVID-19: Hospital Design Strategies to Enhance Patient and Staff Safety

**DOI:** 10.3390/ijerph18168391

**Published:** 2021-08-08

**Authors:** Nirit Putievsky Pilosof, Michael Barrett, Eivor Oborn, Galia Barkai, Itai M. Pessach, Eyal Zimlichman

**Affiliations:** 1 Cambridge Digital Innovation—CJBS & Hughes Hall, University of Cambridge, Cambridge CB1 2EW, UK; 2 Centre for Digital Built Britain, University of Cambridge, Cambridge CB3 0FA, UK; m.barrett@jbs.cam.ac.uk; 3 Cambridge Judge Business School (CJBS), University of Cambridge, Cambridge CB2 1AG, UK; 4 Warwick Business School, University of Warwick, Coventry CV4 7AL, UK; eivor.oborn@wbs.ac.uk; 5 Sheba Medical Center, Tel Hashomer, Ramat Gan 52621, Israel; Galia.Barkai@sheba.health.gov.il (G.B.); Itai.Pessach@sheba.health.gov.il (I.M.P.); Eyal.Zimlichman@sheba.health.gov.il (E.Z.); 6 Sheba BEYOND, Sheba Medical Center, Tel Hashomer, Ramat Gan 52621, Israel; 7 Sackler Faculty of Medicine, Tel-Aviv University, Tel-Aviv 6997801, Israel; 8 Sheba’s Talpiot Medical Leadership Program, Tel Hashomer, Ramat Gan 52621, Israel

**Keywords:** inpatient telemedicine, healthcare design, patient safety, staff safety, control room, model of care, COVID-19

## Abstract

The challenges of the COVID-19 pandemic have led to the development of new hospital design strategies and models of care. To enhance staff safety while preserving patient safety and quality of care, hospitals have created a new model of remote inpatient care using telemedicine technologies. The design of the COVID-19 units divided the space into contaminated and clean zones and integrated a control room with audio-visual technologies to remotely supervise, communicate, and support the care being provided in the contaminated zone. The research is based on semi-structured interviews and observations of care processes that implemented a new model of inpatient telemedicine at Sheba Medical Center in Israel in different COVID-19 units, including an intensive care unit (ICU) and internal medicine unit (IMU). The study examines the impact of the diverse design layouts of the different units associated with the implementation of digital technologies for remote care on patient and staff safety. The results demonstrate the challenges and opportunities of integrating inpatient telemedicine for critical and intermediate care to enhance patient and staff safety. We contribute insights into the design of hospital units to support new models of remote care and suggest implications for Evidence-based Design (EBD), which will guide much needed future research.

## 1. Introduction

The COVID-19 pandemic has driven healthcare institutions to rapidly develop new models of care. The need to protect medical personnel from contamination while providing quality care for COVID-19 patients has led to innovative strategies integrating hospital design with digital technologies for remote care. The new model of inpatient telemedicine evolved from the electronic intensive care unit (e-ICU), which has demonstrated its potential to enhance care [1,2] and its feasibility across diverse settings as a response to the COVID-19 pandemic [3]. While patient safety has always been a major concern in hospital design, telemedicine technologies increased focus on staff safety and the inter-relations between the safety of patients and staff. This study examines the integration of hospital design strategies with the implementation of digital technologies for remote care and the impact of the new model on patient and staff safety across different levels of care.

### 1.1. Hospital Design Strategies for Patient and Staff Safety

Hospital design strategies aim to enhance patient and staff safety by implementing evidence-based design (EBD) for best practice. EBD, defined as “*the process of basing decisions about the built environment on credible research to achieve the best possible outcomes*” [4], was developed following the practice of evidence-based medicine (EBM) that integrates individual clinical expertise with the best available evidence from systematic research [5]. The origin of EBD in healthcare environments goes back to Nightingale’s environmental theory [6] and to the pioneering study of Roger Ulrich on the effects of a view of nature on patient healing [7]. Their work enhanced the development of research in the field of healthcare design and environmental psychology and promoted post-occupancy evaluation (POE) of hospitals to assess the performance characteristics of the design [8]. Many scientific studies have collected empirical evidence demonstrating connections between the environmental design of healthcare facilities and outcomes important for patients, families, staff, and healthcare organisations [9]. Since the publication of the Institute of Medicine “*To Err Is Human”* in 1999, which raised the profile of patient safety and stimulated interventions to prevent hospital-acquired infections and medication errors [10,11], many studies have demonstrated how hospital design can improve patient and staff safety through environmental measures that reduce hospital-acquired infections, medical errors, patient falls, and staff injuries [12,13].

### 1.2. Telemedicine for Patient and Staff Safety

The COVID-19 pandemic has forced all healthcare systems, hospitals, and clinics to rapidly implement telemedicine services [14] and overcome technical and logistic restraints, organisational resistance, and psychological barriers [15]. A key reason that telemedicine solutions have been adopted during the pandemic has been to enable work without physical presence at the workplace, to protect both patients and healthcare professionals. As such, telemedicine has had a positive effect on patient safety and outcomes [16,17]. Research on patient safety in telemedicine settings has demonstrated high levels of diagnosis appropriateness and decision reasonableness, good sensitivity, and high specificity in pediatric tele-triage services [18]. Telemedicine approaches allowed staff in quarantine or in high-risk groups (older, immunosuppressed) to work remotely during the surge [14]. In addition to remote home care, telemedicine was found to be ideally suited to meet the demands of inpatient care while at the same time reducing virus transmission, stretching human and technical resources, and protecting patients and healthcare workers in the inpatient care setting [3,19,20]. Inpatient telemedicine provided a solution to avoid physical contact with COVID-19 infected patients that significantly increases the chance of illness transmission and the need to quarantine exposed healthcare workers. Studies show that virtual pulmonologist or intensivist service (Tele-ICU) allows specialists to remotely manage intubated patients. Physicians are able to see the ventilator settings, patients’ work of breathing and remotely consult with the bedside team while decreasing exposure risk and preserving personal protective equipment [14].

### 1.3. Integration of Telemedicine Technologies with the Hospital Built Environment

New models of inpatient telemedicine for COVID-19 have introduced a new approach to patient and staff safety. The US Facility Guidelines Institute has specified that spaces used for telemedicine communications should be designed to provide patients’ privacy, safety, quality of care, and patient experience, as expected for the same communication taking place in person [21]. Other researchers also argue that telemedicine is not about technology but about people. To support quality of care, they suggest adopting a user-centred design methodology in the development of telemedicine systems [22]. The integration of the built environment of the hospital with digital technologies for remote care and an accelerated global trend provides an opportunity to reassess concepts of telemedicine and EBD in relation to patient and staff safety. As such, examining the manner in which the sudden implementation of inpatient telemedicine during COVID-19 transformed the inter-relationship of patient and staff safety is important to explore.

## 2. The Case of Sheba Medical Center in Israel

The Sheba Medical Center (MC) at Tel HaShomer, the largest tertiary hospital in Israel, rapidly developed inpatient telemedicine units during the COVID-19 outbreak [23]. The new model of care, designed for minimal physical contact with an objective to preserve a high quality of care, was implemented in different medical units, including a COVID-19 Intensive Care Unit (ICU) and COVID-19 Internal Medicine Unit (IMU). The COVID-19 ICU was constructed in only a few days in an underground parking lot, originally designed to serve as a fortified emergency hospital for non-ICU-level patients in times of war [24], and the COVID-19 IMU was constructed in an adapted healthcare building used for geriatric care. The design of the two units divided the space into a contaminated zone and a clean zone using double-door vestibules for the donning and doffing of personal protection equipment (PPE) and split air systems. The site was equipped with special infrastructure for audio and visual communication devices. The new design also included a special control room in the clean zone with telemedicine devices to remotely supervise, communicate, and control the operations in the contaminated zone.

**Figure 1:**
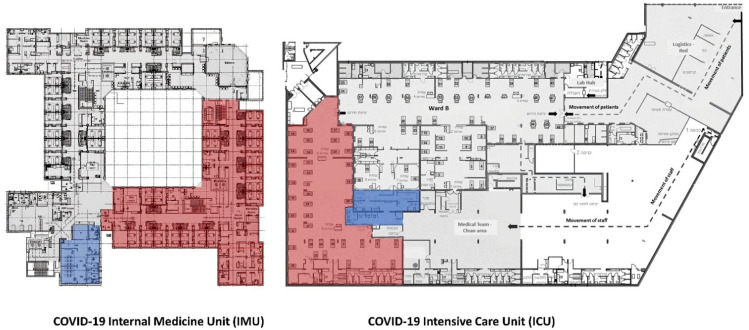
Architectural plans of the COVID-19 IMU (**left**) and the COVID-19 ICU (**right**) illustrating the clinical contaminated zone (in red) and the control room in the clean zone (in blue).

The COVID-19 ICU with an area of 1100 sq. m. (11,840 sq. ft.) has a clinical open space in the contaminated zone for 40 patients, and an adjacent clean zone control room with sealed glass windows between the zones. While there was no direct passage from the clean control room to the contaminated zone, the adjacent location provided a visual sight between the two zones. The COVID-19 IMU with an area of 1400 sq. m. (15,070 sq. ft.) has a reverse L shape layout with semi-private patient rooms for 36 patients. The control room is located in the clean zone near the entrance of the unit with no direct physical sight of the clinical area in the contaminated zone (Table 1, Figure 1).

To support remote patient care, Sheba MC employed different technologies through its ARC innovation Center. The objective was to reduce staff infection risk, upscale care, and lessen errors related to working in protective gear, through constant audio-visual communication between teams in the contaminated zone and the clean control room. The technologies for audio-visual communication, physical examination, monitoring, and management included a video camera on each patient, spatial video cameras, and mobile InTouch telepresence robot [25] in the ICU.

To manage patient and staff safety, the medical staff was divided into two rotating teams: one team working in the contaminated zone with PPE and the second team supporting them from the clean zone control room using remote telemedicine technologies. The objective was to reduce infection exposure of the staff by having minimal staffing inside the contaminated zone, while recognising the necessary bedside care of physicians, nurses, and support personnel for critically ill patients. The divided teams, therefore, rotated between zones every three hours during their twelve-hour shift; three hours in the clean zone, and then three hours in the contaminated zone. The frequent rotation aimed to reduce staff fatigue and challenges of concentration when working with PPE [26].

## 3. Materials and Methods

The exploratory case study involved qualitative inquiry, aiming to analyse the new model of inpatient telemedicine and examine the stakeholders’ interpretive understanding of its impact on the patient and staff safety. The case study received approval from the institutional review board at the Sheba MC in Israel as part of a broader research project studying the smart hospital’s strategic development and planning. Sheba MC provided the first author access to the collected data in collaboration with co-authors from the hospital. The case study was conducted from March to December 2020, based on forty formal interviews with Sheba medical staff, telemedicine experts, and the architectural design team, four days of observations at the COVID-19 ICU and IMU, and guided tours of the COVID-19 units by the Sheba Director of the COVID-19 division and the Sheba Director of infrastructure and building.

The formal one-on-one interviews were semi-structured, including topics such as the use of telemedicine technologies, the impact of the built environment, and the change in professional practice, including support for staff and patients’ safety. The interview questions addressed the development of the new model of care, the personal experience of the staff working in the COVID-19 units, and the challenges and opportunities for future development beyond the crisis. While the topics of the interviews were the same, the questions focused on the specific role of each interviewee. The volunteer subjects for the interviews were selected purposefully by consultation with the hospital management. The participants included sixteen physicians, nine nurses, two human experience directors, three IT administrators, six architects and engineers, and four telemedicine startup directors. The participants included the medical and nursing directors of the units, senior and intern physicians and nurses working in the COVID-19 units, and the supporting teams from the hospital management, IT, and construction divisions. Most of the interviews were held in person in the hospital, based on work availability, and some were conducted virtually by Zoom due to curfew restrictions. Each interview took approximately 30 to 60 min, most being recorded in Hebrew (with the consent of the interviewees), transcribed, and professionally translated into English under the supervision of the researchers. The transcripts were checked with the original recordings for research validity. For analysis, interview notes and field observations were recorded. Additional data included analysis of the architectural plans, hospital webinars, and media coverage. The case study was presented to the hospital board of directors to receive their information and comments.

Based on principles of naturalistic inquiry [27] and a grounded approach to conceptual development [28], thematic qualitative data analysis was adopted to identify emerging themes from the interviews and observations. Following the data collection process, interview transcripts were separated into analytical themes. All the themes were read through twice before coding. Two authors worked through the coded themes and obtained full agreement on themes related to patient and staff safety. Data was organised manually on a spreadsheet. Related and similar ideas were clustered together through the field notes and coding of interviews, eliciting supporting quotes as evidence for the case analysis [29]. The emerging themes were characterised and divided by their impact on the safety of patients and the safety of staff in the COVID-19 ICU and IMU. The data segments within these thematic clusters were then assessed by a third member of the research team to ensure data agreement and accuracy. At the end of the data analysis phase, the validity of the themes was checked and confirmed with clinical leaders with oversight of the care processes. Tables 2 and 3 provide more details of these themes, specifying specific challenges related to COVID-19, the impact of hospital design, the use of digital technologies, and implications of inpatient telemedicine for safety (Tables 2 and 3). While the study presents the implementation of inpatient telemedicine in two different units: ICU and IMU, the study did not aim to compare their patient-related medical outcomes. The two units represent different medical practices, patients in diverse medical conditions, different occupancy rates, length of stay (LOS), and staff ratio. The analysis of the data collection of the two units was presented to the co-authors and medical directors at Sheba MC to have their input on the results and discuss the future implications of the research.

## 4. Results: The Impact of Inpatient Telemedicine on the Safety of Patients and Staff

The new model of remote care at the Sheba MC COVID-19 units transformed practices of inpatient care. The use of digital technologies for remote supervision and management of care in relation to the physical environment of the different units impacts the practice of patient and staff safety. The study investigates the main aspects of patient safety in the COVID-19 units, including (1) prevention of hospital-acquired infection, (2) supervision and monitoring, and (3) reduction of medical errors; as well as the main aspects of staff safety, including (4) prevention of staff contamination with COVID-19, (5) teamwork and collaboration, and (6) management of operations.

The study’s findings indicate the challenges of patient and staff safety in the COVID-19 units and the use of telemedicine technologies to overcome them. The study points out the impact of the units’ design on the implementation of inpatient telemedicine, and the limitations in using telemedicine technologies for the different safety categories. The study also illustrates differences in the use of telemedicine technologies in the ICU and the IMU and specifies safety implications for future design. Our findings are summarised in Table 2 (COVID-19 ICU) and in Table 3 (COVID-19 IMU).

### 4.1. Patient Safety

The safety of patients hospitalised in the COVID-19 ICU and IMU was significantly challenged. In addition to being treated for coronavirus, a disease with limited knowledge regarding effective treatment and risk of fast deterioration, patients’ safety was at risk due to treatment of limited staff working in PPE, high occupancy rates, and lack of family support [30]. To maintain patient safety, the new model of remote care was used to prevent hospital-acquired infections, supervise, and monitor patient conditions, and reduce medical errors.

#### 4.1.1. Prevention of Hospital-Acquired Infections

Telemedicine technologies were used to prevent hospital-acquired infections in addition to COVID-19. The staff was challenged to practice infection control while working with PPE. *“Between one patient and the next, we had to put another robe on over the PPE, which was very hard to do. When you tried to put the extra robe on top of the full PPE, the robe didn’t really protect you. It was all so cumbersome and complicated”* (Nurse in the ICU). The staff also indicated that the built environment affected their practice of infection control. In the open space of the ICU, where patient beds were two meters apart, the staff had to pay special attention to remember to change the extra cover, while in the IMU, the move from one patient room to the other enhanced the practice of changing covers. *“When you go from room to room, you disinfect your hands. When you just move from patient to patient, without any physical barrier, there is nothing to remind you”* (Senior nurse in the ICU).

To maintain patient safety, the staff in the control room watched if the staff in the contaminated zone were changing their additional cover and gloves when moving from patient to patient and alerted them in case they forgot. *“In a situation like this, when you know you’re protected with full PPE, it’s very hard to remember to practice infection control between patients, and for this the video was significant”* (Medical Director in the ICU). The audio-visual technologies became a reminding (nudge) mechanism to prevent infections by punctuating spaces, serving as virtual barriers between patient beds. However, the system was dependent on constant surveillance of staff in the control room, looking at multiple screens to oversee the behavior of the staff in the contaminated zone.

#### 4.1.2. Supervision and Monitoring

The new model of inpatient telemedicine aimed to maintain and even improve the quality of care by remote supervision and monitoring of patients’ conditions. The challenge of monitoring patients by minimal staff working in PPE in the contaminated zone was overcome by constant remote monitoring of patient conditions by staff in the control room. The remote monitoring was used to make clinical decisions: “*If the remote monitor provides all the clinical measurements, and I can focus the camera and see the patient from a clinical standpoint, I don’t have to be inside the unit*” (Medical Director in the IMU); and for nursing supervision: “*You can focus the camera up close to the respirator and it’s as if you are standing next to the screen at the bedside. You see the data, you see the patient, you can see how much urine is in his bag. All of that is from within the control room, and you don’t have to bother the nurse inside*” (Nurse in the ICU). The system was also used to oversee patient’s movement in the IMU to alert them in case of risk of falls.

The remote supervision enhanced the staff’s sense of control. “*When I sit in the control room, I feel I have more control then when I work in the nurse station inside, because there I can’t hear and can’t see (with the PPE), and I don’t know what is happening with the patients*” (Nurse in the IMU). The staff also reported the advantages of watching over different patients simultaneously on the screens, which increased equality of care for patients disregarding their location in the unit. “*When I’m looking at one patient, I also grasp what’s happening with someone else. It’s important. It gives a much better control* “(Head nurse in the ICU). Still, the constant remote supervision of patients was challenging for the staff in the control room. The segregated monitoring systems and camera views on multiple screens required concentration and orientation. “*It’s very hard to constantly watch all the patient rooms, especially as we do not have more staff. One person cannot do it. It is very complicated*” (Senior doctor in the IMU).

The constant supervision of patients by video cameras, intended to enhance the quality of care, denied patients’ privacy and at certain times compromised personal dignity. The patients had limited control over their exposure and data privacy, without knowledge of who is watching on the other side. As research regarding mental health consequences of isolated hospitalised patients with COVID-19 showed high rates of depression, anxiety, and post-traumatic stress symptoms (PTSS) [31,32], the approach of remote supervision should consider not only patients’ physical safety but also its impact on their psychological safety.

#### 4.1.3. Reduction of Medical Errors

Medical errors were a big concern in COVID-19 units due to new medical protocols, minimal staff at the bedside working with PPE, and less experienced staff recruited to work in the COVID-19 units. *“…You’re dressed in that overall, and you can’t see while there are life-threatening situations and patients are receiving medication in incredible amounts like I never saw before*” (Senior nurse in the ICU). Staff reported a significant decrease of abilities when working in protective gear. “*The ability to see the wider picture, the ability to physically concentrate, the ability to receive information from the equipment, your ability to communicate with advisors, which is something very important in ICU. All of that was significantly impaired*” (Medical Director in the ICU).

To prevent medical errors, telemedicine technologies were used to supervise and substitute, as much as possible, the work of staff in the contaminated zone. “*Technology was used to improve the level of care and prevent caregivers from spending a lot of time next to the patient, based on the understanding being that long period of time next the patient is difficult, affects concentration and ability and most likely increases the chance of mistakes in staff’s activity and care of the patient*” (Senior nurse in the ICU). Telemedicine technologies were also used “*to make sure that staff who are caring for such complex patients for the first time are doing things properly. From the control room, I could focus on the medication a particular patient was receiving and see what was written there”* (Senior nurse in the ICU). Remote supervision and communication were also used to guide interns by experienced doctors and nurses working in the control room. “*The audio-visual technologies afforded the director and specialists, medics and nurses, to share their expertise without the discomfort of PPE*” (Medical Director in the ICU). Yet, the remote supervision of medical treatment was constrained by the limitations of virtual visibility dependent on the camera’s height, angle, and ability to zoom in relation to the location of activity in the physical space.

### 4.2. Staff Safety

The safety of staff working in the COVID-19 units was a major concern for the hospital organisation. Prevention of staff contamination with COVID-19 was crucial to maintain the workforce and enhance staff’s sense of security. The health and wellbeing of staff became central to the organisation’s resilience, recognising the need to focus not only on physical safety but also on mental health safety [33,34,35,36,37,38]. As such, the new model of remote care by digital technologies was developed to protect staff, physically and mentally, by preventing staff contamination with COVID-19, supporting bedside caregivers working in PPE, and providing a management tool for critical events.

#### 4.2.1. Prevention of Contamination with COVID-19

The main purpose of inpatient telemedicine was to prevent staff contamination with COVID-19. As opposed to regular times when the main objective is the health of patients, in the COVID units, the health of staff was considered equally important. “*Safeguarding the lives of staff was elevated to the same level as safeguarding the lives of patients*” (Medical Director in the IMU). Since staff presence at patient beds is required, remote care was used to reduce the number of staff inside. “*Although it was an intense full-time nursing care, the use of technologies and the fact that the ward was built with an external control room, enabled us to lessen the number of staff inside*” (Telemedicine Director). This strategy was proven effective as staff exposures were lower than in non-COVID units in the hospital [23]. The new model of remote care was also developed to address the staffs’ fear of contamination. “*Most of the staff at the beginning were scared of the patients. That didn’t exist in the past, and it’s a big change. There was always the possibility of becoming infected by disease, but corona brought this fear to a whole new level. It was similar to the situation with HIV in the beginning, when people were so scared*” (Telemedicine Program Developer). Inpatient telemedicine provided the staff a sense of security which led to a shift in the professional conception toward telemedicine. “*The fear of becoming infected and the desire to work without the PPE, and on the other hand, the strong will to safeguard our ability to provide care to our patients, caused us to use telemedicine*” (Medical Director).

In addition to reducing the number of staff working in the contaminated zone, telemedicine technologies allowed to supervise the infection control of the staff to protect themselves. The staff in the control room watched the staff in the contaminated zone and alerted them if there was any concern with their PPE. “*I was using the robot to wander around the room, going from bed to bed, and all of a sudden, I saw a doctor lift his hand, and the entire seam of his robe was torn… He didn’t notice. No one noticed. In a normal situation, when I do my rounds surrounded by 5-6 doctors, I would not have noticed a tear in a doctor’s robe, I would be looking at the patient, not at the doctors*” (Medical Director in the ICU).

#### 4.2.2. Teamwork and Collaboration

The limited staff working in the contaminated zone covered with PPE were dependent on the support of their peers in the control room. Collaboration and teamwork contributed not only to the physical safety of the staff, but also to their mental health and emotional wellbeing. The staff in the control room supported the bedside caregivers, acting as their “eyes and ears”. “*In the big area of the ICU, you don’t always hear equipment that beeps, especially when wearing all the PPE, you don’t always recognise what’s going on. The job of the nurse sitting in the control room was to call us if they identified a monitor that was beeping on the screen and tell us which monitor it was on which bed*” (Nurse in the ICU).

Inpatient telemedicine transformed professional practices to support bedside caregivers. “*We simply created an additional arm of remote care that was meant to allow staff to work without the PPE and enable them to do procedures that were very hard to do with the PPE*” (Medical Director). The staff performed all possible work remotely in the clean zone using digital technologies, including equipment organisation, medicine preparation, and clinical documentation. “*We did not want the nurses to work on computers in the infected area, so we developed a method for remote documentation of patient’s treatment by telemedicine*” (Medical Director). The new model also shifted the collaboration of doctors and nurses working together. “*In the regular unit, if I saw something that wasn’t right, I had to go to the doctor, look for him in his office, tell him what I saw, maybe he has to go to see the patient. Here, we sit together, examine the data, and right away the doctor says what to do*” (Senior nurse in the ICU).

Digital technologies were a key mediator enabling the segregation of the staff in the two zones. Yet, the ease of communication was described as essential for remote teamwork. “*The bedside caregiver cannot leave the patient and walk over to the communication equipment. The equipment has to be accessible, one centimeter from him. That is why we used Motorola in the end. If we could speak with a specific person through earphones, it would be great, but it was problematic with the PPE*” (Medical Director in the ICU).

#### 4.2.3. Management of Operations

The management of the unit was responsible for deciding on patient care and staff operation remotely. The lack of medical staff and experts in the contaminated zone required a strategy for remote coordination of staff, especially during critical events. The physical layout of the units had a significant impact on the ability to manage events. “*The IMU is built in an L shape, so if a doctor is at one end of the floor and something happens at the other end, you have to be able to speak to him quickly and get him from one place to the other*” (Medical Director in the IMU). The division to patient rooms compared to the open space also created a challenge for managing limited staff in the contaminated zone. “*If there are two patients in two different rooms and both are unstable, one central team will go from one to the other and if I try to call, they are in the room and they can’t hear the phone on the counter at the nurse station*” (Nurse in the IMU).

Digital technologies, which offered audio-visual communication to locate the staff and prioritise the work, increased the ability to control operations and maintain staff efficiency. The new model of care also provided opportunities for remote consultation with experts in the hospital, which contributed not only to the safety of patients but also to the safety of the staff by enhancing their sense of control. However, remote management depends on advanced communication and spatial awareness of situations. “*One of the things that the camera does, is it divides the whole into parts, creating a challenge in the control of space… the director needs to know who is next to which bed, where the equipment is, where something is happening. In a video, when you have 40 beds, you don’t immediately catch on that something specific is happening*” (Medical Director). For this purpose, the integration of virtual visibility with physical visibility through the window between the control room and the contaminated zone in the ICU enhanced the control of operations even further. “*The window allowed us to see what was happening in the open space. You can see the whole dynamic. The staff inside might be totally concentrating on what is happening in one place, but we could see all of the other patients. If you are only watching them on a camera, you can’t go from one screen to the next to have an overview of the whole unit*” (Nurse in the ICU).

## 5. Discussion

The study explored the implementation of telemedicine for inpatient care in COVID-19 ICU and IMU’s, focusing on the integration of digital technologies within the hospital-built environment and its impact on patient and staff safety. Previous research on the replacement of telemedicine for in-person care has shown that safety is not compromised in different medical fields [18,39,40]. However, our findings suggest that the integration of the system for hospital inpatient care requires a holistic approach. Such approaches would develop work systems that not only include the physical environment, the people (patient, staff, families), and the tasks performed, but also integrate the tools and technology used, as well as the organisational policies, which work together for achieving safe patient care [41]. Further, our research findings show how digital technology use is intimately connected to the level of care and the design of the medical units, while the practices that develop around these technologies are significant in promoting patient and staff safety (Table 2 and Table 3).

The new model of inpatient telemedicine, developed initially for staff safety to prevent staff contamination with COVID-19, minimising the need to traverse physical distance (for example, between clean and contaminated zones), also promoted patient safety. In both units, patient safety was enhanced in three ways, though the practices around these varied with spatial arrangement and level of care specific to the unit (Table 1, Figure 1). First, safety was potentially enhanced by providing staff with tools to help adhere with infection control best practices. Hospital-acquired infections are an ongoing concern [42] which became more problematic during COVID-19 due to new practices around PPE use and the need for cumbersome layers of gloves and gowns. While staff were well protected behind these layers, the new practices around changing outer gloves and gowns between patients were difficult to sustain, with few tactile reminders. Digital technologies enabled remote staff to remind colleagues from a distance of the need to change gloves, adding an additional protective reminder to prevent the spread of infections between patients. Second, safety for patients was augmented through new ways to supervise and monitor. This was particularly important for patients who were more distant from care staff or control rooms. Staff shortages during the pandemic more generally challenged the supervision of patients, particularly when occupancy rates were high. Being able to scan a number of screens simultaneously, noting if there was a fall or vital signals flashing, added safety through the technology’s ability to bring together multiple spaces. Third, patient safety was enhanced by potentially decreasing medical errors. Working around COVID-19 precautions and unfamiliar treatments meant staff were frequently adjusting to new protocols of care. Safety was augmented as technologies provided new mechanisms to support learning and training. In particular, technology use occasioned that medical experts and experienced staff could provide guidance and support to those less experienced, such as trainees, with the new procedures.

Our study also revealed three ways that staff safety was enhanced. First, staff safety was enhanced by minimising the contamination of staff from the infectious virus. The contagion had been a primary reason for installing digital technologies, with safety being achieved as staff could now work from a distance in safer zones. However, staff in contaminated zones also benefitted as colleagues were able to spot rips or tears in PPE and flag the exposure from their unique digital vantage points. During the COVID crisis, technology functioned as a digital safety barrier from the virus—a “digital PPE” [26]. Second, safety was enhanced for staff through new communication patterns and enabling teamwork. The physical and mental exhaustion of wearing PPE hindered morale, and the typical encouragement staff would provide for each other. An increased sense of isolation put staff at risk, particularly in terms of mental health, however, digital technology was able to reintegrate effective human contact, though distant, raising awareness between staff of each other, and providing opportunities for faceless trust to develop by making expressions visible. Third, staff safety was enhanced through improved operational management. Improved coordination between distant others was particularly important during sudden incidents or emergencies. Digital technologies provided environmental awareness, enabling users to scan the rooms and hallways to identify operational challenges and coordinate rapid responses.

At the same time, our study highlights two unexpected concerns that emerged with the use of technology for staff safety. First, the use of telemedicine with its many monitoring (camera) systems can fragment the visibility and this can lead to emotional labor for already stretched staff. The unevenness of multiple (dis)connected images are not always easily assimilated in a timely manner for effective monitoring and designing the screen arrangements may be important for gaining a more holistic understanding, thereby facilitating effective supervision. These concerns for patient and staff safety are magnified during high occupancy levels and during busy periods or where spatial layouts are complex. Second, there is a need to consider the dynamic relationship between patient and staff safety as unintended consequences can arise. With inpatient telemedicine, new work routines and PPE use formed a boundary between staff and patients, which shifted the focus to staff safety and lessened the traditional emphasis on patient engagement and contact. As such, overcompensating for staff safety can unintentionally come at the expense of patient-centred care.

## 6. Conclusions

The COVID-19 crisis disrupted conceptions of healthcare services and presented an opportunity to develop and test new models of hospital inpatient care. The integration of digital technologies for remote care within the built environment of the hospital created a new model of care that enhanced patient and staff safety. While the model was developed specifically for COVID-19 units, the study points to the potential of developing inpatient telemedicine beyond the challenges of the current crisis. The approach can support healthcare systems dealing with a lack of staff [43] by providing flexibility in staffing enhanced by remote consultations and manage changes in patient loads by offering tools to supervise and monitor more patients without compromising patient and staff safety. Inpatient telemedicine can also augment infection control and improve space utilisation overcoming limitations of distance and remote location by providing a systematic approach to hospital operations. Developing insights from inpatient telemedicine, with attention on patient and staff safety, can inform methods for home hospitalisation with supervision by the hospital. This approach can help support future transformations of the healthcare eco-system integrating hospital care with more remote locations such as the community and home care.

The study illustrates possible implications for future hospital design. Virtual visibility of patients and staff holds the potential to transform previous conceptions of healthcare design, focusing on providing direct visibility by optimising the unit’s layout [44,45,46,47]. Remote care can also transform conceptions of nurse station design [48,49] into a central control room, questioning its location and relation to the clinical area and to other units [50]. The potential to increase equality of care for patients regardless of their location in the unit, can advance the development of hybrid models of inpatient and outpatient care. Concurrently, the study also specifies implications for the future development of telemedicine technologies. The challenge of using multiple digital technologies for patient monitoring and audio-visual communication on segregated screens points to a need to develop one comprehensive platform to support the multi-purpose use of the technologies. The necessity of spatial awareness of where patients, staff, and equipment are located in the unit points to the potential to develop a digital twin to represent the dynamic movement of users for real-time operations.

Future development of inpatient telemedicine beyond COVID-19 requires consideration of the fundamental conceptions of inpatient hospital services that were challenged during the crisis, including patient-centred care, direct physical observation, human connection, and family involvement. The need to protect staff and enhance patient safety has led to practices that were not previously considered appropriate. Constant video capture of patients and staff with the reduced human touch of bedside caregivers, while patients become isolated without family support as occurred during the COVID-19 pandemic raises significant challenges regarding patient and staff privacy, patient control, and human experience. The advancement of inpatient telemedicine should address these issues in the design of the built environment and the specifications of how remote digital technologies are applied.

While this study introduces an innovative solution for inpatient telemedicine during the outbreak of COVID-19, further work is needed to compare and evaluate different solutions to enhance patient and staff safety more permanently using digital technology. More studies on the implementation of telemedicine technologies in different layouts of hospital units are required, as well as various medical specialties and models of care. Further research on the impact of inpatient telemedicine on the design of healthcare facilities in diverse environmental, cultural, and economic contexts will enhance the knowledge base needed for the future development of healthcare architecture and digital technologies for remote care.

## Figures and Tables

**Table 1:** Key Features of inpatient telemedicine in the COVID-19 units at the Sheba Medical Center.

Key Features	Intensive Care Unit (ICU)	Internal Medicine Unit (IMU)
Level of care	Critical care	Intermediate care
Layout of the unit (Figure 1)	Square shape–open space	Reverse L shape–single corridor
Number and type of patient beds/rooms	40 patient beds with movable privacy curtains	36 patients in 18 semi-private patient rooms
Area of the unit	1100 sq. m. (11,840 sq. ft.)	1400 sq. m. (15,070 sq. ft.)
Location of the control room	Adjacent to the unit	Near the entrance to the unit
Relation between the control room and the unit	Panoramic window that provides direct visibility from both sides	No direct visibility
Location of cameras in the clinical contaminated zone	Throughout the open ward and near all patient beds	Central nurse station and in all patient rooms
Audio-visual technologies	Video camera per patient, spatial video cameras, walkie-talkie, InTouch telepresence robot	Video camera per room, spatial video cameras, walkie-talkie
Communication between users	Staff to staff, staff to patients, staff to family	Staff to staff, staff to patients, staff to family, patients to family

**Table 2:** The impact of inpatient telemedicine on patient and staff safety in the COVID-19 ICU.

COVID-19 Intensive Care Unit (ICU)				
Safety Category	Safety Challenges due to COVID-19	The Impact of Hospital Design	The Use of Telemedicine	Safety Implications
Dimension of PATIENT SAFETY				
Prevention of hospital-acquired infections	Practicing infection control wearing an extra robe and gloves on top of the PPE when moving between patients	The open space with no barriers between patient beds hinder the practice of infection control	Supervision of staff in ensuring infection control by video cameras and audio communication	Inpatient telemedicine affords flexibility of space while minimizing prevention of infections
Supervision and monitoring	Critical care patients in need of constant supervision while PPE limits the abilities of staff	High-occupancy rates of patient in beds scattered in the large space of the unit limits staff visibility of patients	Virtual visibility and remote monitoring of patient condition to alert in case of deterioration	Inpatient telemedicine augments the level of supervision in high occupancy rates and large units
Reduction of medical errors	New medical protocol, minimal staff at the bedside, challenge to consult with PPE, less experienced staff	Lack of natural light in the underground environment and noise in the open space increase risk	Supervision of staff performing medical protocols, remote guidance of staff and interns by more experienced staff	Inpatient telemedicine supports staff competence while working in changing conditions
Dimension of STAFF SAFETY				
Prevention of Contamination with COVID-19	Need for constant bedside care of critical patients while protecting staff from exposure to the coronavirus	Division to clean and contaminated zones with separated air systems and circulation routes	Remote care of patients from the control room with a minimal number of staff working in the contaminated zone	Inpatient telemedicine supports intensive care for patients in isolation while protecting staff
Teamwork and collaboration	Physical and mental challenge of working with PPE, lack of competence and sense of control	Visibility of staff through the window in the control room enhance teamwork and collaboration	Audio-visual communication to support and help the staff in the contaminated zone working with PPE	Inpatient telemedicine enhances collaboration among staff working in detached spaces
Management of operations	Lack of staff and experts in the contaminated zone to treat multiple critical events	Visibility of staff and equipment in the open space supports situation awareness and management	Audio-visual communication to prioritize and manage the staff in case of multiple critical events	Inpatient telemedicine increases control of operations and efficiency of staff in case of emergency

**Table 3:** The impact of inpatient telemedicine on patient and staff safety in the COVID-19 IMU.

COVID-19 Internal Medicine Unit (IMU)				
Safety Category	Safety Challenges due to COVID-19	The Impact of Hospital Design	The Use of Telemedicine	Safety Implications
Dimension of PATIENT SAFETY				
Prevention of hospital-acquired infections	Practicing infection control wearing an extra robe and gloves on top of the PPE when moving between patients	Semi-private rooms increase risk of hospital-acquired infections between patients within the room	Supervision of staff and patient’s behavior to ensure infection control by audio-video communication	Inpatient telemedicine affords increase of capacity in patient rooms while moderating infection risks
Supervision and monitoring	Complex patients in need of constant supervision while PPE limits the abilities of staff	The L shape layout of the unit limits the visibility of patients in distant rooms from the nurse station	Virtual visibility of rooms to alert in case of emergency. Communication with Family for supervision of care.	Inpatient telemedicine augments the level of supervision of patients disregarding their location in the unit
Reduction of medical errors	New medical protocol, minimal staff at the bedside with PPE, less experienced staff	Semi-private rooms and distance between rooms increase the risk	Supervision of medicine distribution and remote guidance of staff and interns by more experienced staff	Inpatient telemedicine reduces risk of medical errors in dynamic and complex systems
Dimension of STAFF SAFETY				
Prevention of contamination with COVID-19	Need for constant supervision of COVID-19 patients while protecting staff from exposure to the coronavirus	Multi circulation routes provide separation between movement of COVID-19 patients and staff	Supervision of patient and staff movement in and out of the unit to alert in case of contamination risk	Inpatient telemedicine supports the movement of patients in isolation while protecting staff from infection
Teamwork and collaboration	Physical and mental challenge of working with PPE, lack of competence and sense of control	Distance and location of patient rooms reduces collaboration and teamwork	Audio-visual communication to support and help the isolated staff working in different rooms with PPE	Inpatient telemedicine supports staff competence while working alone in patient rooms
Management of operations	Lack of staff and experts in the contaminated zone to treat multiple critical events	The L shape layout with patient rooms limits the visibility of staff and equipment and decreases situation awareness	Audio-visual communication to locate and manage the staff in case of multiple critical events	Inpatient telemedicine increases situation awareness and control of operations in case of emergency
